# Technology of Bacteria Investigation

**Published:** 1886-01

**Authors:** 


### BOOK REVIEWS.

THE TECHNOLOGY OF BACTERIA INVESTIGATION. *Explicit directions for the study of Bacteria, their culture, staining mounting, etc., according to the methods employed by the most eminent investigators. By* CHARLES S. DOLLEY, M. D. *Boston:* S. S. CASSINO & CO. *1885. Chicago:* W. T. KEENER.

It could hardly be expected that Dr. Dolley, in so small a work, would exhaust the subject he has endeavored to treat. In his preface he states that his work is put forth with the view of stimulating careful study of schizomycetes by American investigators.

He has conveniently grouped his material under three heads: I. General Directions ; II. Special Methods for Investigating Pathogenic Bacteria ; III. Formulary. Under Part I. he reviews the most approved methods of obtaining and studying micro-organisms; (a) in the living state; (b) fixed, hardened, and stained,—including an allusion to the photographic branch of bacterial study. The author treats very exhaustively of the different modes of cultivation,—devoting, however, surprisingly little space to vaccination and inoculation experiments. Part I. closes with some remarks on what the writer styles "Biological Analysis," in which there is nothing new or especially interesting.

In Part II. the different recognized specific micro-organisms in disease are described, but we regret that the material collected on such subjects as malaria, diphtheria, syphilis, and yellow fever is so meagre. The specific poisons of scarlatina, measles, meningitis, etc., are merely alluded to.

In Part III., viz., *Formulary*, the directions for the preparation of staining-fluids, culture-media, instructions as regards mounting, specimens, etc., are very full and clear, making the book a valuable one for the laboratory.

The work is placed before the public in an excellent form, but we regret that it is devoid of illustrations, for in no subject, we believe, are illustrations more necessary. We believe it to be a book which the profession needs, and which will fully repay any one who will read what the author has so carefully collected and systematically grouped. At the end of each section, references are given to the different authorities, making it very easy to go back to the original sources of information.

